# Intrafractional prostate motion during external beam radiotherapy monitored by a real‐time target localization system

**DOI:** 10.1120/jacmp.v16i2.5013

**Published:** 2015-03-08

**Authors:** Xu Tong, Xiaoming Chen, Jinsheng Li, Qianqian Xu, Mu‐han Lin, Lili Chen, Robert A. Price, Chang‐Ming Ma

**Affiliations:** ^1^ Radiation Oncology Department Third‐Affiliated Hospital of Qiqihar Medical University Qiqihar China; ^2^ Radiation Oncology Department Fox Chase Cancer Center Philadelphia USA

**Keywords:** 4D localization, intrafractional motion, prostate cancer, radiation therapy

## Abstract

This paper investigates the clinical significance of real‐time monitoring of intrafractional prostate motion during external beam radiotherapy using a commercial 4D localization system. Intrafractional prostate motion was tracked during 8,660 treatment fractions for 236 patients. The following statistics were analyzed: 1) the percentage of fractions in which the prostate shifted 2−7 mm for a certain duration; 2) the proportion of the entire tracking time during which the prostate shifted 2−7 mm; and 3) the proportion of each minute in which the shift exceeded 2−7 mm. The ten patients exhibiting maximum intrafractional‐motion patterns were analyzed separately. Our results showed that the percentage of fractions in which the prostate shifted by >2,3,5,and 7 mm off the baseline in any direction for >30 s was 56.8%, 27.2%, 4.6%, and 0.7% for intact prostate and 68.7%, 35.6%, 10.1%, and 1.8% for postprostatectomy patients, respectively. For the ten patients, these percentages were 91.3%, 72.4%, 36.3%, and 6%, respectively. The percentage of tracking time during which the prostate shifted >2,3,5,and 7 mm was 27.8%, 10.7%, 1.6%, and 0.3%, respectively, and it was 56.2%, 33.7%, 11.2%, and 2.1%, respectively, for the ten patients. The percentage of tracking time for a >3 mm posterior motion was four to five times higher than that in other directions. For treatments completed in 5 min (VMAT) and 10 min (IMRT), the proportion for the prostate to shift by >3 mm was 4% and 12%, respectively. Although intrafractional prostate motion was generally small, caution should be taken for patients who exhibit frequent large intrafractional motion. For those patients, adjustment of patient positioning may be necessary or a larger treatment margin may be used. After the initial alignment, the likelihood of prostate motion increases with time. Therefore, it is favorable to use advanced techniques (e.g., VMAT) that require less delivery time in order to reduce the treatment uncertainty resulting from intrafractional prostate motion.

PACS number: 87.50.S‐, 87.53.Kn, 87.55.N‐, 87.55.ne

## I. INTRODUCTION

In recent years, the development of advanced therapy techniques, such as intensity‐modulated radiation therapy (IMRT) and volumetric‐modulated radiation therapy (VMAT), has facilitated novel treatment strategies employing dose escalation and hypofractionation schemes in the management of prostate cancer. An important issue in prostate radiotherapy is the toxicity of the rectum and bladder, which can be improved by the accurate localization of the treatment target and the reduction of the treatment margins. The displacement and deformation of the prostate and surrounding organs are important factors in the consideration of treatment margins for external radiotherapy of prostate cancer.^1^, ^2^, ^3^ The effects of inter‐ and intrafractional motion and deformation of the treatment target during prostate radiotherapy have been investigated using various imaging techniques in order to develop practical margin recipes for commonly available treatment setup and target localization techniques. The application of advanced patient setup and online target location techniques allows for the use of smaller treatment margins to reduce normal tissue toxicities.

Many techniques, such as ultrasound, infrared cameras, X‐ray imaging, in‐room CT, kilovoltage and megavoltage cone‐beam CT (CBCT), rectal balloon, and magnetic resonance imaging, have been used to investigate inter‐ and intrafractional prostate motion.^4^, ^5^, ^6^, ^7^, ^8^, ^9^, ^10^, ^11^, ^12^, ^13^, ^14^, ^15^ Translational and sometimes rotational adjustments can be made to the treatment target before the treatment is delivered. X‐ray imaging has been used successfully to measure intrafractional motion, allowing for real‐time target localization and tracking. However, patients will receive additional radiation due to repeated exposures, especially for long treatment procedures. The Calypso 4D localization system (Calypso Medical Technologies, Seattle, WA) uses miniaturized, nonionizing implanted fiducial devices,^16^, ^17^, ^18^ which are electromagnetic transponders, called Beacons, to set up the patient efficiently and to track the location of the treatment target during external beam radiotherapy. This will allow for accurate localization of the treatment target both before and during the treatment, and real‐time tracking of the target during treatment. The system has received 510K clearance from the U.S. Food and Drug Administration (FDA) for prostate and lung radiotherapy.

A number of investigations have been reported on the use of the Calypso system for prostate real‐time tracking during external beam radiotherapy.^16^, ^17^, ^18^, ^19^, ^20^, ^21^, ^22^, ^23^, ^24^ However, some early studies suffered either from a limited number of patients or from selected treatment fractions, which did not provide sufficiently large patient datasets for systematic analyses of intrafractional prostate motion patterns. For example, based on 550 Calypso records of 17 patients, Langen et al.^(20)^ reported that the proportion per minute for the prostate to shift by >5 mm was 6% at the 11th minute. However, for one patient, 100% of all 11th min intervals was spent at a displacement >5 mm. It will be more useful to know what percentage of a regular patient population will exhibit such large prostate motion and benefit from real‐time motion tracking. Based on the results of 35 patients, Kupelian et al.^(18)^ reported that approximately 15% of fractions (83% of patients) exhibited a 5 mm shift for cumulative 30 s. However, detailed analyses showed that the majority of patients did not exhibit substantial prostate movements in any fractions, while some patients had up to 56% of fractions with >5 mm shifts for >30 s. It will be clinically important to identify such patients with substantial prostate movements and design “personalized” treatment strategies for them.

In our institution, both IMRT and VMAT have been applied in prostate radiotherapy, which requires different beam‐on times to deliver the same prescription doses. It is expected that prostate treatments that require longer beam‐on times will suffer more from intrafractional prostate motion if its effects cannot be corrected during treatments. We have been using the Calypso system for prostate treatment setup and intrafractional motion correction for several years. A large number of patients, who were treated with advanced radiotherapy techniques under Calypso 4D real‐time monitoring, have been accrued. In this study, we analyzed a total of 8,660 treatment records from 236 Calypso patients who were treated for the tumor bed or the intact prostate with and without seminal vesicles, to investigate intrafractional prostate motion patterns. Ten prostate patients with the largest prostate displacement during the entire course of treatment were further analyzed to investigate the worst‐case scenario and their prostate motion patterns are reported in detail.

## II. MATERIALS AND METHODS

### A. The Calypso 4D localization system

The Calypso system uses three electromagnetic transponders (Beacons) implanted in the prostate gland (or surrounding tissues for postprostatectomy patients) to facilitate treatment setup and target localization. A panel is positioned above the patient, which houses a magnetic array that induces the Beacons and accepts the resonance signals sent back by the Beacons, to allow for the detection and continuous monitoring of the locations of the three Beacons relative to the isocenter of the treatment machine, at a frequency of 10 Hz. In order to reduce the effects of prostate edema caused by the Beacon implantation and possible Beacon migration, CT scans were performed a week after the Beacon implantation. MR scans were used to facilitate target and critical structure delineation and they were performed before the Beacon implantation to avoid artifacts in the MR images caused by coils in the Beacons.

The Calypso system uses the centroid of the three Beacons to track the treatment target location during dose delivery, and it also reports changes in the relative Beacon positions in the initial treatment setup so that a judgment can be made as to whether a Beacon has migrated relative to the others or significant deformation has occurred. The prostate rotation information is also included in the treatment report, but it was not used in the target localization process because the treatment couch only allows for translational movements and there may be large uncertainties in the rotation angle calculation due to even minor Beacon migration and/or prostate deformation, which cannot be determined independently with the Calypso system.^(25)^

During a treatment setup, the patient was first aligned with skin marks and lasers and then the Calypso system was used to precisely align the treatment plan isocenter (related to the centroid of the Beacons) with the machine isocenter by necessary couch shifts, which set the baseline position for the subsequent monitoring. Weekly CBCT was performed after the Calypso alignment to confirm the Beacon locations and their relationship to the prostate geometry to ensure the accuracy for prostate localization. The differences in the prostate anatomy and Beacon positions between the simulation CT and the CBCT were used to determine the migration of any Beacons and its effect on the relationship of the treatment plan isocenter and the centroid of the Beacons. The Calypso system allows the use of one or two Beacons to localize the target if any Beacons have migrated. The prostate localization accuracy for treatment setup was similar between the Calypso system and CBCT/Beacons (estimated to be 2 mm), while the strength of the Calypso system was to track the target movement during treatment.

Displacement of the treatment target (i.e., the centroid of the Beacons) relative to the baseline was continuously monitored during dose delivery, and treatment was interrupted if the displacement in any direction exceeded 5 mm. A couch shift was made to realign the patient back to the baseline position, and the treatment continued. In our institution, a 5 mm posterior margin was used to build the PTV, while an 8 mm margin was used for other directions for both intact prostate and prostatectomy patients. A 5 mm shift threshold was used in the real‐time monitoring process to ensure the accurate dose delivery to the target, while the relative movement between the prostate (bed) and the seminal vesicles/lymphatics was considered by the 8 mm CTV to PTV margins. For all treatment fractions analyzed in this work, about 10% of the treatment fractions required realignment and, therefore, the recorded absolute centroid positions also included the applied couch shifts. For this study, the couch shift values for individual fractions were recovered to reproduce the actual prostate displacement data during the entire treatment periods without the prostate position intervention.

### B. Patients and data analysis

A total of 8,660 Calypso treatment records from 236 patients who were treated with the segmental (i.e., step‐and‐shoot) IMRT technique were randomly selected. Among them, 200 were treated for intact prostates (total 7,738 treatment records) and 36 for postprostatectomy radiation (total 922 treatment records). For some patients, some treatment fractions were not monitored with the Calypso system due to its occasional downtime; instead, the treatment setup and target localization were guided with CBCT/Beacons. All treatments were carried out on two Varian Trilogy accelerators using 10 MV photon beams (Varian Medical Systems, Palo Alto, CA). For postprostatectomy patients, the prescription dose was 64−68 Gy, while for intact prostate patients, the prescription dose was 76−80 Gy to the prostate and proximal seminal vesicles and 56 Gy to the distal seminal vesicles and lymphatics, if included. All treatments were delivered at 2 Gy per fraction. The number of treatment records per patient ranged from 30 to 40; each recorded the prostate movement in three major directions (left–right, anterior–posterior, and inferior–superior) from completion of the treatment setup to the last beam‐off during a treatment fraction. The treatment time as recorded for the 8,660 fractions lasted 3.8−25 min, and the average treatment time was about 8.0±3.9 min (1σ).

The implantation and simulation procedures have been published previously^(26)^ and are briefly described below. Patients were instructed to come to all simulations and treatments with an empty rectum and a full bladder. All patients were simulated and treated in the supine position with a customized thermoplastic foam cast for immobilization and positioning. On the day of implantation, an MRI simulation was performed before the Beacon placement procedure. Three radiofrequency Beacons were implanted into the prostate (bed) under ultrasound guidance. One week later, a noncontrast CT simulation was obtained and the CT images were fused with the MR images. At simulation, the treatment isocenter was placed within the prostate (bed) and, guided by lasers aligned to the simulator isocenter, triangulation tattoos are marked on the skin surface. The treatment target, bladder, rectum, femoral head, and small bowel volumes were determined and contoured following the RTOG postoperative prostate volume guidelines.^(27)^ The three Beacons were identified and contoured on the simulation CT and were designated right, left, and apex Beacons, according to their relative positions. The locations of the beacons on the simulation CT defined a baseline set of coordinates for the Calypso system to determine target isocenter shifts for daily localization and tracking.

In order to investigate the intrafractional prostate movement and its potential effects on IMRT and VMAT with different treatment durations, we analyzed the 8,660 Calypso treatment records according to the percentage of treatment fractions during which the prostate was displaced by more than 2, 3, 5 or 7 mm in any direction relative to the initial setup position for a cumulative time >10 and >30 s, respectively. Although we corrected the prostate location when its shift reached 5 mm, we have recovered the 7 mm shift information from our measured data to help understand the trend of the prostate movement, which may be useful to other investigators in their margin determination without the use of a real‐time monitoring system. We also evaluated the intrafractional motion patterns based on the percentage of the total treatment time (including all treatment fractions) during which the prostate was displaced by more than 2, 3, 5 or 7 mm from its baseline position. Patients treated for prostate beds or intact prostates were analyzed separately. As previous investigations have reported that 4%–5% of prostate patients exhibited significant intrafractional prostate motion, we further analyzed ten prostate patients with the largest prostate displacement during the entire course of treatment to investigate the worst‐case scenario. Finally, we analyzed the trends of intrafractional prostate displacement as a function of treatment time after completion of the treatment setup, and the number of fractions during which significant prostate displacements in any direction occurred in the first minute. Statistical analysis was performed for the measured movements. The mean and standard deviation of the mean (SEM) were calculated and the results were expressed as mean ±SEM. To determine if there was a significant difference between different groups, Student's *t*‐test was used and a p‐value, p≤0.05, was considered to be statistically significant.

## III. RESULTS

### A. Qualitative observations

The intrafractional prostate movements were generally small (i.e., <2 mm), but could be substantial (e.g., >5 mm) for a small number of patients. From the 8,660 Calypso records, we observed the main trends of intrafractional prostate movement such as occasional fast shifts (e.g., due to muscle contraction, within seconds), short‐term shifts (e.g., due to gas passage, within several to tens of seconds), continuous displacement (e.g., due to rectal/bladder filling), and the combination of various movements. The least movement occurred in the lateral direction, and the anterior and superior shifts were often occasional or short‐term due to gas passage and sometimes correlated with each other. The posterior and inferior displacements generally occurred together, and were often continuous, slow movements, which were likely caused by bladder filling.

### B. Intrafractional motion vs. time

**Figure 1:**
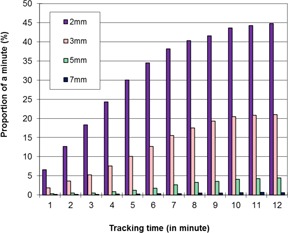
The proportion of each minute in which the prostate displacement was more than 2, 3, 5, or 7 mm in any direction. For this plot, the prostate displacement for the first, second, and each of the subsequent minutes from all tracking fractions was analyzed separately.

**Figure 2:**
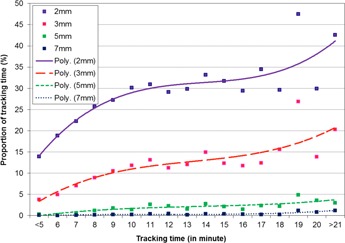
The proportion for the prostate to shift more than 2, 3, 5, or 7 mm as a function of the total tracking time. For this plot, the prostate displacement for the first 5 min, and each of the subsequent minutes of the whole tracking time was presented together with a polynomial fit.

We first investigated the prostate intrafractional displacement as a function of treatment time for the entire patient population studied in this work. Figure 1 shows the percentage of the time when the prostate shifted >2,3,5,and 7 mm during each minute after treatment setup for the entire treatment duration. It is evident that by the fifth minute, the proportion for the prostate to shift >3 mm was 10% (per minute), while by the tenth minute the proportion for the prostate to shift >3 mm exceeded 20% (per minute). Figure 2 shows the percentage of treatment time for the prostate to displace >2,3,5,and 7 mm for treatment fractions that were completed within 5, 6, and up to 20 min after the treatment setup. The percentage of the accumulated time for the prostate to shift >3 mm over the total treatment time was 4% if the treatment was completed within 5 min, and this percentage increased to 12% for all treatment fractions that were completed within 10 min. However, it was also observed that, for some treatment fractions, the prostate started substantial drifts almost immediately after the treatment setup; among the 8,660 treatment fractions studied there were 951, 269, 59, and 21 fractions during which the prostate shifted by >2,3,5,and 7 mm for longer than 10 s, respectively, within the first minute. There were 39 fractions during which the prostate shifted >3 mm within seconds after the therapist pressed the button to set the baseline, while there were 14 fractions during which the prostate shifted >5 mm within 30 s after the baseline was set.

### C. Intrafractional motion vs. direction

We then investigated the intrafractional motion in individual directions. Table 1 shows the percentage of the total treatment time during which the treatment target (i.e., the centroid of the Beacons) shifted >2,3,5,and 7 mm in each direction including the maximum, the minimum, the average, and the standard deviation for the 8,660 treatment records. It is evident that there was little movement in the left and right directions. The most significant displacement was in the posterior direction; the cumulative time of the posterior displacement for >3 mm was 6.4% of the total treatment time, while it was 1.7%, 1.5%, and 1.3%, respectively, in the superior, inferior, and anterior directions. From Table 1 it can be seen that, for some patients, the target intrafractional motion was >5 mm in the posterior direction for the entire treatment time (the maximum is 100%), while it was more than 7 mm in the superior and anterior directions for almost 80% of the treatment duration (the maximum is 79.3%). It should be mentioned that any intrafractional displacements >5 mm were corrected by couch adjustments during the treatment at our institution with the use of the Calypso system. The results shown here are the actual target intrafractional movements with the couch adjustments recovered and edited out.

**Table 1:** Percentage of the total tracking time during which the treatment target was displaced by more than 2,3,5,or 7 mm, respectively, in each direction. The values are expressed as the mean (minimum−maximum)±SEM (1 SD) for the 8,660 treatment fractions

	*Treatment Target Displacement*			
*Direction*	>2 mm	>3 mm	>5 mm	>7 mm
Left	0.7 (0−100)±6.3	0.2 (0−100)±2.8	0 (0−12.1)±0.2	0 (0−2.1)±0
Right	0.7 (0−100)±5.3	0.1 (0−98.1)±1.5	0 (0−15.5)±0.3	0 (0−9.1)±0.1
Superior	5.7 (0−100)±15.7	1.7 (0−93.0)±7.6	0.3 (0−84.6)±2.8	0.1 (0−79.3)±1.6
Inferior	4.8 (0−100)±14.6	1.5 (0−100)±7.9	0.1 (0−72.6)±2.1	0 (0−6.4)±0.2
Anterior	3.0 (0−100)±10.7	1.3 (0−94.6)±6.1	0.4 (0−85.8)±3.3	0.1 (0−79.3)±1.7
Posterior	17.0 (0−100)±26.7	6.4 (0−100)±16.1	0.8 (0−100)±5.7	0 (0−43.8)±0.7

### D. Intact prostate vs. prostate bed

Because of the difference in the anatomical structure, patients with intact prostates are expected to show different intrafractional movements compared to those who received postprostatectomy radiotherapy. The data in Table 2 show that the percentage of treatment fractions during which the prostate intrafractional movement was >2,3,5,and 7 mm and the cumulative time was >10 s in any direction relative to the baseline was 65.2%, 34.1%, 8.2%, and 2.3%, respectively, for intact prostate patients. If the cumulative time increased to 30 s, the percentage decreased to 56.8%, 27.2%, 4.6%, and 0.7%, respectively. For patients receiving postprostatectomy radiotherapy, the corresponding percentages were 75.6%, 42.8%, 15.2%, and 4.7% for a >10 s cumulative time, and 68.7%, 35.6%, 10.1%, and 1.8% for a >30 s cumulative time, respectively. The differences between the intact prostate and prostatectomy patients are all statistically significant (p<0.01). For the worst ten patients who exhibited the largest prostate/tumor bed displacement, the percentage of treatment fractions during which the prostate/tumor bed shifted by more than 2, 3, 5, and 7 mm was 95.2%, 76%, 43.2%, and 14.7% for a >10 s accumulation, and 91.3%, 72.4%, 36.3%, and 6% for a >30 s accumulation, respectively. Again, these values are statistically different from those for the general population (p<0.01).

For some patients, the intrafractional displacement was slow and continuous while, for others, it was random and transitional. It would be useful to know whether shorter treatment times are beneficial for some patients, or larger treatment margins are required for others. Our results showed that for patients with intact prostates, the percentage of the total treatment time during which the prostate shifted by more than 2, 3, 5, and 7 mm was 27.0%, 10.1%, 1.4%, and 0.2%, respectively, while for postprostatectomy patients this percentage increased to 34.0%, 14.7%, 2.7%, and 0.6%, respectively (Table 3). This means that the prostate intrafractional motion is generally small for this patient population as a whole. For the ten patients whose prostate displacement was the largest, however, the percentages were 56.2%, 33.7%, 11.2%, and 2.1%, respectively, which certainly deserves special attention in the determination of margin recipes for treatments with shorter beam‐on times and fewer fractions. Table 4 shows the percentage of fractions and percentage of the entire treatment time during which the target shifted substantially for a certain duration for the ten worst patients; all of them showed >5 mm target shifts in more than 30% of their fractions. A detailed investigation also revealed that all of them exhibited >3 mm drifts in most fractions during the first week and >5 mm drifts in at least 2 fractions during the first two weeks (results not shown).

**Table 2:** Percentage of treatment fractions during which the prostate or the tumor bed was displaced by more than 2, 3, 5, or 7 mm off the baseline in any direction for at least 10 and 30 s. All the paired values between intact prostate and prostatectomy are statistically different (p<0.01)

	*% Fractions for* >10 s		*% Fractions for* >30 s	
*Treatment Target Displacement*	*Intact Prostate*	*Postprostatectomy*	*Intact Prostate*	*Postprostatectomy*
>2 mm	65.2	75.6	56.8	68.7
>3 mm	34.1	42.8	27.2	35.6
>5 mm	8.2	15.2	4.6	10.1
>7 mm	2.3	4.7	0.7	1.8

**Table 3:** Percentage of the total tracking time during which the prostate or the prostate bed was displaced by more than 2,3,5,or 7 mm in any direction. All the paired values between intact prostate and prostatectomy are statistically different (p<0.01)

	*Percentage of Total Treatment Time*			
*Direction*	>2 mm	>3 mm	>5 mm	>7 mm
Intact Prostate	27.0%	10.1%	1.4%	0.2%
Postprostatectomy	34.0%	14.7%	2.7%	0.6%

**Table 4:** The percentage of fractions and the percentage of entire treatment time during which the target shifted more than 3,5,or 7 mm for longer than 10 and 30 s for the ten worst patients (five intact prostate and five postprostatectomy)

	*Percentage of Fractions*						*Percentage of Treatment Time*		
	>10 s			>30 s					
*# of fractions*	>3 mm	>5 mm	>7 mm	>3 mm	>5 mm	>7 mm	>3 mm	>5 mm	>7 mm
40	70.0%	45.0%	10.0%	67.5%	40.0%	0.0%	38.1%	12.2%	0.3%
40	87.5%	42.5%	5.0%	87.5%	37.5%	0.0%	47.6%	16.2%	0.1%
39	76.9%	38.5%	7.7%	74.4%	33.3%	0.0%	34.8%	8.9%	0.2%
39	64.1%	30.8%	17.9%	59.0%	30.8%	15.4%	34.1%	14.2%	9.9%
39	64.1%	30.8%	15.4%	56.4%	23.1%	2.6%	23.6%	9.3%	1.0%
31	96.8%	67.7%	29.0%	87.1%	51.6%	16.1%	41.4%	15.9%	4.2%
30	80.0%	56.7%	26.7%	73.3%	46.7%	10.0%	24.1%	10.6%	3.9%
33	54.5%	33.3%	18.2%	54.5%	24.2%	9.1%	18.1%	7.9%	4.1%
34	76.5%	32.4%	8.8%	76.5%	29.4%	2.9%	34.8%	8.9%	0.2%
33	72.7%	30.3%	3.0%	63.6%	27.3%	0.0%	33.8%	8.4%	0.1%

## IV. DISCUSSION

In this work, a total of 8,660 treatment records were analyzed, which represented the largest amount of intrafractional motion data ever published in the literature as far as we know. Therefore, it would be interesting to compare our findings with those published previously by other investigators. Our results showed that the percentages of fractions during which the prostate targets shifted by >3 mm and >5 mm for a cumulative time >30 s were 27% and 5%, respectively, which were less than those reported by Kupelian et al.^(18)^ (41% and 15%, respectively) based on 1,157 Calypso records of 35 patients for intact prostate patients. They also reported that about 1 in 12 fractions required realignment of the patient after the prostate moved by more than 5 mm. In our work, we found that about one in ten intact prostate patients and one in five postprostatectomy patients required realignment after they exhibited >5 mm shifts. Our proportion for the prostate to shift by >3 mm was 10% at the fifth minute and 20% at the tenth minute, while the corresponding results reported by Langen et al.^(20)^ based on 550 Calypso records of 17 patients were one in eight and one in four, respectively. The percentage of time for the prostate to shift >3 mm and >5 mm in our work was 10.7% and 1.6% for both intact prostate and postprostatectomy patients, respectively, which was slightly less than the percentages reported by Li et al.^(19)^ based on 775 Calypso records of 105 patients (13.4% and 1.8%, respectively). Other techniques^9^, ^11^, ^14^, ^15^ have been used to investigate intrafractional prostate motion and most reported intrafraction motion with respect to maximum displacements and their standard deviations within a small number of patients. For instance, Padhani et al.^(14)^ reported ≥5 mm anterior–posterior displacements in 29% of 7 min cine MR scans for 55 patients, and Malone et al.^(15)^ reported ≥4 mm vertical displacements in 8% and ≥4 mm longitudinal displacements in 23% of the fractions for 28 patients, using fluoroscopy and implanted markers. As pointed out by Kupelian et al.,^(18)^ however, the time factor (i.e., how long the prostate remains beyond a certain threshold) is probably important, because some displacements might be short‐lived and clinically inconsequential. Only continuous, real‐time motion data can provide such information.

It is reasonable to assume that prostate intrafractional motion generally increases with the treatment time and therefore advanced treatment techniques that employ shorter times to deliver the same prescription doses would be more advantageous. Based on the data of this study, by the fifth minute the accumulated time for the prostate to shift by >3 mm was 10% of the observation time (per minute) for the entire patient population investigated in this work. In contrast, the accumulated time for the prostate to move >3 mm by the tenth minute after the Calypso setup exceeded 20% of the observation time (per minute). In our institution, both IMRT and RapidArc have been used for prostate treatments. However, only IMRT was used for Calypso patients because the IMRT delivery could be resumed after the interruption of the treatment to reset the isocenter, while early versions of the RapidArc did not allow for the continuation of an arc if it was stopped during the delivery. Based on the analysis of 20 RapidArc patients treated in our institution, the average beam‐on time was 2.57 min (at least two arcs were used in our prostate treatment planning to ensure the quality of the treatment plans, and the beam‐on time was the total duration from the beginning of the first arc to the end of the second arc). Together with the additional imaging time to check the Calypso setup (e.g., weekly validation of the relative Beacon's locations to the treatment anatomy) and the time for the therapists to walk out of the room and initiate the treatment, the entire RapidArc delivery can be completed in 5 min for most RapidArc patients. For IMRT treatments, the average beam‐on time (from the beginning of the first MLC field to the end of the last MLC field) was 6.49 min for up to 9 gantry angles/ports without carriage shifts (based on the IMRT patients investigated in this work). Together with the additional imaging for setup validation and the time for the therapists to walk out of the room and initiate the treatment, most of these IMRT treatments could be completed in 10 min. For IMRT patients with 10–18 MLC fields (mostly due to large fields that had to be split into two on the Varian accelerator and required a carriage shift) the average beam‐on time increased to 10.78 min and many treatments would need 13–15 min to complete routinely. This indicates that the cumulative time for the prostate to move >3 mm for IMRT patients would be more than twice as that for RapidArc patients (both were treated on the Varian accelerators and planned on the Varian Eclipse treatment planning system).

Interestingly, we also observed substantial prostate drifts of >3 mm in 269 fractions and >5 mm in 59 fractions within the first minute after the treatment setup, which occurred randomly among patients. These were likely due to the patient's physiological and psychological preparations/adjustments for the treatment (e.g., muscle relaxation, position adjustments, rectal gas release after the therapists left the room). Therefore, it may be useful to observe the prostate displacement for 1 min after the treatment setup and then decide whether or not to begin dose delivery, especially if the beam‐on time is only a few minutes, which may be affected even more by short‐term prostate displacements (e.g., those caused by gas passage).

Since the minimal (posterior) margin used in this work to build the PTV was 5 mm, a 5 mm shift threshold was used during treatment delivery and the isocenter location was reset if the prostate shifted by 5 mm in any direction. For the ten patients who exhibited the largest prostate displacements, the percentage of time for the prostate to shift >5 mm was 11.2%. This implies that, during this time, a part of the prostate CTV would have been outside of the intended treatment volume if we had not reset the treatment isocenter based on real‐time monitoring. Since many prostate patients presented demonstrable disease in the peripheral zone, significant underdosing could result from long periods of time when the prostate gland moved out of the planned dose distribution for some of those fractions. Our results showed that the accumulated time for the prostate to shift posteriorly by >5 mm was much longer than that in other directions, which was consistent with previous findings.^21^, ^24^ For the ten patients who exhibited most prostate displacements in this work, the percentage of cumulative time for the prostate to shift >5 mm was 7.4%; the dose received by the posterior portion of the prostate was as low as 1.6 Gy instead of the planned 2 Gy for some of the worst fractions. These results were consistent with the reports of Chen et al.^(28)^ who reconstructed the dose distributions of prostate patients based on CT‐on‐rails images taken before and after IMRT treatments.

It is worth noting that although the majority of our Calypso patients did not exhibit clinically significant prostate intrafractional motion during the entire treatment course, this was only confirmed with the use of the Calypso system (i.e., one could not tell which patient would exhibit large intrafractional prostate displacement without actually monitoring it with the Calypso system). On the other hand, it could be more efficient to use the Calypso system for the first one or two weeks to identify those patients who might exhibit large intrafractional motion frequently and to continue applying real‐time monitoring only if it might bring practical benefits. An alternative method to identify the small group of patients who often have large prostate intrafractional motion may be to perform CBCT scans before and after the radiation delivery for one or two weeks, then apply different margin recipes to those patients if large intrafractional displacements are observed.^(9)^

Finally, it should be mentioned that we only analyzed the prostate intrafractional movements in the six major directions, which were monitored and corrected using translational couch movements if they were greater than the preset (5 mm) thresholds. Due to abdominal pressure, the filling of the bladder and/or rectum, and other factors, the prostate may deform or rotate during the course of the treatment. The Calypso system reported the changes of relative Beacon locations, which might be caused by prostate deformation and/or Beacon migration, and the prostate rotation angles, which might be greatly affected by the relative Beacon locations.^(25)^ Prostate rotations were not corrected because of the limited couch movements. The effects of the prostate intrafractional deformation and rotation were not included in this work, and will be explored in future studies.

V. CONCLUSIONS

The intrafractional displacement of the prostate is very small (<2 mm) for most patients, but some patients may exhibit either frequent or continuous substantial (>5 mm) prostate movement, which will require large treatment margins to ensure accurate dose delivery. The Calypso real‐time localization system has been used for precise treatment setup and to reduce the effects of intrafractional motion with preset intervention thresholds; the treatment can be interrupted and the effects of the prostate displacement can be corrected by couch shifts. Some patients may show short‐term shifts immediately after the treatment setup. As the treatment time increases, the intrafractional prostate displacement will generally increase. Therefore, the treatment margins may be set based on the treatment time required to complete the dose delivery. Some advanced techniques such as VMAT/RapidArc may be favorable in this regard to reduce the treatment time needed for the same prescription doses.

## References

[1] Langen KM and Jones DT . Organ motion and its management. Int J Radiat Oncol Biol Phys. 2001;50(1):265–78. 11316572 10.1016/s0360-3016(01)01453-5

[2] Kitamura K , Shirato H , Seppenwoolde Y , et al. Three‐dimensional intrafractional movement of prostate measured during real‐time tumor‐tracking radiotherapy in supine and prone treatment positions. Int J Radiat Oncol Biol Phys. 2002;53(5):1117–23. 12128110 10.1016/s0360-3016(02)02882-1

[3] Li HS , Chetty IJ , Enke CA , et al. Dosimetric consequences of intrafraction prostate motion. Int J Radiat Oncol Biol Phys. 2008;71(3):801–12. 18234439 10.1016/j.ijrobp.2007.10.049

[4] Lattanzi J , McNeeley S , Pinover W , et al. A comparison of daily CT localization to a daily ultrasound‐based system in prostate cancer. Int J Radiat Oncol Biol Phys. 1999;43(4):719–25. 10098426 10.1016/s0360-3016(98)00496-9

[5] Scarbrough TJ , Golden NM , Ting JY , et al. Comparison of ultrasound and implanted seed marker prostate localization methods: implications for image‐guided radiotherapy. Int J Radiat Oncol Biol Phys. 2006;65(2):378–87. 16563658 10.1016/j.ijrobp.2006.01.008

[6] Herman MG , Pisansky TM , Kruse JJ , Prisciandaro JI , Davis BJ , King BF . Technical aspects of daily online positioning of the prostate for three‐dimensional conformal radiotherapy using an electronic portal imaging device. Int J Radiat Oncol Biol Phys. 2003;57(4):1131–40. 14575846 10.1016/s0360-3016(03)00766-1

[7] Nederveen A , Lagendijk J , Hofman P . Detection of fiducial gold markers for automatic on‐line megavoltage position verification using a marker extraction kernel (MEK). Int J Radiat Oncol Biol Phys. 2000;47(5):1435–42. 10889399 10.1016/s0360-3016(00)00523-x

[8] Schallenkamp JM , Herman MG , Kruse JJ , Pisansky TM . Prostate position relative to pelvic bony anatomy based on intraprostatic gold markers and electronic portal imaging. Int J Radiat Oncol Biol Phys. 2005;63(3):800–11. 16199313 10.1016/j.ijrobp.2005.02.022

[9] Deurloo KE , Steenbakkers RJ , Zijp LJ , et al. Quantification of shape variation of prostate and seminal vesicles during external beam radiotherapy. Int J Radiat Oncol Biol Phys. 2005;61(1):228–38. 15629616 10.1016/j.ijrobp.2004.09.023

[10] Soete G , De Cock M , Verellen D , Michielsen D , Keuppens F , Storme G . X‐ray‐assisted positioning of patients treated by conformal arc radiotherapy for prostate cancer: comparison of setup accuracy using implanted markers versus bony structures. Int J Radiat Oncol Biol Phys. 2007;67(3):823–27. 17197124 10.1016/j.ijrobp.2006.09.041

[11] Kotte ANTJ , Hofman P , Lagendijk JJW , van Vulpen M , van der Heide UA . Intrafraction motion of the prostate during external‐beam radiation therapy: analysis of 427 patients with implanted fiducial markers. Int J Radiat Oncol Biol Phys. 2007;69(2):419–25. 17513059 10.1016/j.ijrobp.2007.03.029

[12] Teh BS , Woo SY , Mai WY , et al. Clinical experience with intensity‐modulated radiation therapy (IMRT) for prostate cancer with the use of rectal balloon for prostate immobilization. Med Dosim. 2002;27(2):105–13. 12074461 10.1016/s0958-3947(02)00092-4

[13] Wachter S , Gerstner N , Dorner D , et al. The influence of a rectal balloon tube as internal immobilization device on variations of volumes and dose‐volume histograms during treatment course of conformal radiotherapy for prostate cancer. Int J Radiat Oncol Biol Phys. 2002;52(1):91–100. 11777626 10.1016/s0360-3016(01)01821-1

[14] Padhani AR , Khoo VS , Suckling J , Husband JE , Leach MO , Dearnaley DP . Evaluating the effect of rectal distension and rectal movement on prostate gland position using cine MRI. Int J Radiat Oncol Biol Phys. 1999;44(3):525–33. 10348281 10.1016/s0360-3016(99)00040-1

[15] Malone S , Crook JM , Kendal WS , Szanto J . Respiratory‐induced prostate motion: quantification and characterization. Int J Radiat Oncol Biol Phys. 2000;48(1):105–09. 10924978 10.1016/s0360-3016(00)00603-9

[16] Balter JM , Wright JN , Newell LJ et al. Accuracy of a wireless localization system for radiotherapy. Int J Radiat Oncol Biol Phys. 2005;61(3):933–37. 15708277 10.1016/j.ijrobp.2004.11.009

[17] Willoughby T , Kupelian P , Pouliot J , et al. Target localization and real‐time tracking using the Calypso 4D localization system in patients with localized prostate cancer. Int J Radiat Oncol Biol Phys. 2006;65(2):528–34. 16690435 10.1016/j.ijrobp.2006.01.050

[18] Kupelian P , Willoughby T , Mahadevan A , et al. Multi‐institutional clinical experience with the Calypso system in localization and continuous, real‐time monitoring of the prostate gland during external radiotherapy. Int J Radiat Oncol Biol Phys. 2007;67(4):1088–109. 17187940 10.1016/j.ijrobp.2006.10.026

[19] Li JS , Pollack A , Horwitz EM , Buyounouski MK , Ma CM . Observations of prostate intrafractional motion during external beam radiation therapy. WC 2009. IFMBE Proceedings; 2009;25:499–502.

[20] Langen KM , Willoughby T , Meeks SL , et al. Observations on real‐time prostate gland motion using electromagnetic tracking. Int J Radiat Oncol Biol Phys. 2008;71(4):1084–90. 18280057 10.1016/j.ijrobp.2007.11.054

[21] Curtis W , Khan M , Magnelli A , Stephans K , Tendulkar R , Xia P . Relationship of imaging frequency and planning margin to account for intrafraction prostate motion: analysis based on real‐time monitoring data. Int J Radiat Oncol Biol Phys. 2013;85(3):700–06. 22795802 10.1016/j.ijrobp.2012.05.044

[22] Wilbert J , Baier K , Hermann C , Flentje M , Guckenberger M . Accuracy of real‐time couch tracking during 3‐dimensional conformal radiation therapy, intensity modulated radiation therapy, and volumetric modulated arc therapy for prostate cancer. Int J Radiat Oncol Biol Phys. 2013;85(1):237–42. 22541958 10.1016/j.ijrobp.2012.01.095

[23] Bittner N , Butler WM , Reed JL , et al. Electromagnetic tracking of intrafraction prostate displacement in patients externally immobilized in the prone position. Int J Radiat Oncol Biol Phys. 2010:77(2):490–95. 19775826 10.1016/j.ijrobp.2009.05.033

[24] Li JS , Lin MH , Buyounouski M , Horwitz EM , Ma CM . Reduction of prostate intrafractional motion from shortening the treatment time. Phys Med Biol. 2013;58(14):4921–32. 23798642 10.1088/0031-9155/58/14/4921 PMC3940444

[25] Xu Q , Li J , Shan G , et al. Comparison of prostate rotation and Calypso Beacon rotation for prostate margin evaluation [abstract]. Med Phys. 2009;36(6):2704.

[26] Canter D , Greenberg RE , Horwitz EM , et al. Implantation of electromagnetic transponders following radical prostatectomy for delivery of IMRT. Can J Urol. 2010;17(5):5365–69. 20974028

[27] Michalski JM , Lawton C , El Naqa I , et al. Development of RTOG consensus guidelines for the definition of the clinical target volume for postoperative conformal radiation therapy for prostate cancer. Int J Radiat Oncol Biol Phys. 2010;76(2):361–68. 19394158 10.1016/j.ijrobp.2009.02.006 PMC2847420

[28] Chen L , Paskalev K , Xu X , et al. Rectal dose variation during the course of image‐guided radiation therapy of prostate cancer. Radiother Oncol. 2010;95(2):198–202. 20303193 10.1016/j.radonc.2010.02.023

